# The Diagnosis and Surgical Treatment of Septic Thrombosis of the Lateral Sinus

**Published:** 1892-01-02

**Authors:** 


### THE PRACTITIONER'S MIRROR AND RETROSPECT.

[*The Editor will be glad to receive offers of co-operation and contributions from members of the profession. All letters should be addressed to* THE EDITOR, THE LODGE, PORCHESTER SQUARE, LONDON, W.]

### MEDICINE.

### THE DIAGNOSIS AND SURGICAL TREATMENT OF SEPTIC THROMBOSIS OF THE LATERAL SINUS.

A Septic clot in the lateral sinus has until recently seemed quite beyond the reach of surgery. We could drain the mastoid antrum, and we could even deal with abscess in the brain secondary to ear disease, but to interfere with a clot in a large venous channel did not not seem possible. We knew that portions of the septic clot became detached and formed infarcts in various organs, and yet for a long time it did not occur to anyone to cut off the infarction by ligature of the vein. However, a few years ago Horsley suggested that this should be done; but only when signs of embolism appeared. But why should he wait for embolism to begin? Lane, in 1889, operated on a boy, and he recovered. Ballance,* has lately operated in four cases. In the first case ligature of the vein was followed by marked improvement, and although pyæmic infarction followed, the man recovered. In the second case there was only some temporary improvement. The third patient seemed on the way to recovery when a small abscess formed in the larynx and she died of laryngeal dyspnœa. With regard to the fourth case we had, perhaps, better use Mr. Ballance's own words. He says, "What the operation did for this patient is really marvellous: from a position of imminent danger she was transferred to one of practical safety." On the whole, I think, we may be encouraged by these cases.

But these cases seem to show that ligature of the vein does not wholly stop the risk of infarction: and it seems to us that this may be explained by the communication which takes place between the internal and external jugular vein in the parotid region, and by the possibility of regurgitation of septic material through the inferior petrosal sinus and transverse sinus into the lateral sinus of the other side, as well as at the torcular herophili. Ballance, however, says it is an error to suppose the lateral sinuses are continuous here. He describes the right sinus as continuous with the superior longitudinal sinus, and the left with the straight. But he probably means simply that the currents run as he describes, and we think there must certainly be a possibility of septic clot getting into the lateral sinus of the opposite side.

The communications of the internal with the anterior jugular vein is not very important, but we would suggest that the external as well as the internal jugular vein should be tied in these cases, as an additional hindrance to the dissemination of septic emboli.

But it will be asked, Can the circulation of the part be satisfactorily carried on if the jugular is tied? There is no doubt that it can. Before we operate the sinus is filled with clot, and ligature of the vein lower down, although it causes temporary congestion, is soon followed by compensation.

And it is well not to be content with ligation of the vein. It is best to leave open the upper end in the wound in the neck, and syringe through from the mastoid opening.

The great importance of the surgical treatment of septic thrombosis of the lateral sinus is well illustrated by its frequency. During twenty years, in nearly half the deaths from complications of middle ear disease, at Guy's Hospital, septic thrombosis of the lateral sinus was present.

And where are we likely to find these cases? Not under the care of the surgeon ready labelled for surgical interference, but under the physician, sent in as cases of pneumonia, bronchitis, and enteric fever, because of the pyæmic lung mischief and the high temperature. These are the cases that, again and again, must die without any correct diagnosis being made, and now that they can be treated, how vast does the importance of their early recognition become, and yet how difficult in many cases?

The value of all surgical treatment is based on accuracy of diagnosis, and so before dealing with the treatment of this affection we must discuss the possibility and difficulties of its diagnosis. Complications of middle ear disease are often so mixed up together, and their symptoms are so far from pathognomonic, that diagnosis is often very difficult. A septic process in the middle ear may not only cause thrombosis in the sinus, but also sub-dural abscess or meningitis, or the septic process may cause thrombosis and then spread through the wall of the sinus to the pia mater and cause a secondary meningitis. The symptoms present in some cases which are relieved by merely incising the membrana tympani are so severe as to simulate some complication, *i. e.*, the symptoms produced by pus pent up in the middle ear. Headache, shivering, vomiting, and drowsiness have been produced in this way as well as the pain in the ear which we should expect. This is the more important as rigors are one of the most important signs of a pyæmic process, and yet they may be present in mere retention of pus. How then are we to distinguish the retained pus from the commencement of pyæmia? In many cases we can only watch and see what will happen. If there is no opening through the tympanic membrane we must incise it and wash out. If there is, or if after incising the membrane symptoms still continue, we must open up the mastoid and syringe through. If symptoms still continue we must proceed in a manner presently to be described to deal with the lateral sinus. Fortunately, it is at the very beginning we may be unable to diagnose between these conditions, later on the pyæmic temperature chart, and possibly the signs of extension of clot presently to be described will aid us. We hope in our second paper to go more fully into the differential diagnosis.

Again, we must remember that thrombosis of the sinus and other serious complications of middle ear disease may be present without any otorrhœa, so that their nature is not suspected, and such cases have been diagnosed as typhoid, whereas the post-mortem disclosed their true nature. But on the other hand, neither must we forget that typhoid sometimes, though rarely, begins suddenly with a rigor, and that otorrhœa is occasionally an early complication, as pointed out by Murchison; but in typhoid the temperature does not fall to normal every now and then, as in pyæmia and the rigors are not so often repeated.

What, then, will be asked, are the pathognomonic signs of thrombosis in the sinus? In what cases are we to operate? The only pathognomonic signs are those due to extension of the clot, but they may not be present. There are numerous symptoms which are not pathognomonic, and it is often a combination of these that we have to rely on in our diagnosis. Thrombosis may extend in several directions. It may spread down the internal jugular veins and give rise to one of the most important signs that are ever present, *i.e.*, to swelling and tenderness in its course. Whether this is due to a phlebitis or to enlargement of the deep glands around the veins seems uncertain. Then we may get tenderness and œdema over the mastoid from blocking of the mastoid vein another extension of clot; but this may, we think, be due to the suppuration in the mastoid, and consequently of less

* *Lancet*, Vol. 1., 90. p. 1057.

diagnostic value. However, this local œdema and tenderness may be absent when fœtid pus is present in the groove for the sinus and a septic clot occupies its lumen. Lastly, we may have extension of the clot into the cavernous sinus, causing proptosis and œdema of eyelids and conjunctiva.

Then with regard to symptoms which are not in themselves pathognomonic, but which occurring together strongly point to thrombosis. First and foremost is pyæmia. Although this may be due to other complications, especially to subdural abscesses, or, indeed, to suppuration in the middle ear itself, yet if other symptoms of thrombosis are present, strongly points to it, as so often it is due to dissemination of the septic clot. Ballance attaches much importance to tenderness on deep pressure at some point along the posterior border of the mastoid in the course of the lateral sinus. Vomiting is sometimes a marked symptom, and headache, delirium, and optic neuritis are present sometimes in cases in which, post-mortem, the only complications present were pus in the groove for the sinus and septic thrombosis of the sinus. Stiffness of the muscles at the back of the neck is also sometimes present. But these symptoms are variable and may be present in other conditions, and it seems to us that the swelling and tenderness over the jugular; the tenderness in the course of the lateral sinus; and the presence of pyæmic temperature with rigors are the chief indications.

What were the indications in the four cases Ballance operated on? In the first case there was slight swelling and tenderness over the upper part of the jugular vein, and pyæmia was present. In the second case, besides a history of pain in the head and neck, vomiting, shivering, and delirium at night, there was marked tenderness on deep pressure at the posterior edge of the mastoid, and tenderness and œdema over the upper part of the internal jugular vein. The third case was only diagnosed through a history of repeated rigors with vomiting, in connection with ear disease. The presence of pyæmia, together with the tenderness over the posterior edge of the mastoid and doubtful swelling about the jugular vein enabled the diagnosis to be made in the fourth case, taken in conjunction with other symptoms of less diagnostic value.

Pyæmia from septic thrombosis usually lasts about eighteen days, drowsiness and coma coming on several days before death. The lungs are the chief organs affected, and it is a curious fact that joint troubles are not common. There is often profuse diarrhœa and jaundice from sapræmia, the former, of course, increasing the resemblance these cases have sometimes to typhoid.

Why should we confine this method of treatment to septic thrombosis? Pyæmia may follow suppuration in the middle ear alone, as well as subdural abscess without septic thrombosis in the sinus. Both we can drain through the mastoid; but should we not also (as Mr. Ballance and Dr. Hawkings* suggest, ligature the jugular vein, which must be as much the channel of infection as if it contained a septic clot? True, we cannot remove the carious part of the temporal bone, which originates the suppuration, but neither do we attempt this when we deal with a septic clot or a subdural abscess secondary to such a condition. We can cut off the septic infarction without.

And surely this treatment of pyæmia from ear disease opens to us a far wider field of surgery. Can we do nothing to cut off the supply of infected material in these terribly fatal cases of pyæmia following "acute necrosis" and wounds in the extremities?

We hope in our next paper to describe the method of dealing with these cases of thrombosed septic sinus.

* St. Thomas's Hospital Reports for 1890.

